# Modulating glioma-mediated myeloid-derived suppressor cell development with sulforaphane

**DOI:** 10.1371/journal.pone.0179012

**Published:** 2017-06-30

**Authors:** Ravi Kumar, Tristan de Mooij, Timothy E. Peterson, Tatiana Kaptzan, Aaron J. Johnson, David J. Daniels, Ian F. Parney

**Affiliations:** 1 Department of Neurological Surgery, Mayo Clinic, Rochester, Minnesota, United States of America; 2 Department of Immunology, Mayo Clinic, Rochester, Minnesota, United States of America; University of Alabama at Birmingham, UNITED STATES

## Abstract

Glioblastoma is the most common primary tumor of the brain and has few long-term survivors. The local and systemic immunosuppressive environment created by glioblastoma allows it to evade immunosurveillance. Myeloid-derived suppressor cells (MDSCs) are a critical component of this immunosuppression. Understanding mechanisms of MDSC formation and function are key to developing effective immunotherapies. In this study, we developed a novel model to reliably generate human MDSCs from healthy-donor CD14^+^ monocytes by culture in human glioma-conditioned media. Monocytic MDSC frequency was assessed by flow cytometry and confocal microscopy. The resulting MDSCs robustly inhibited T cell proliferation. A cytokine array identified multiple components of the GCM potentially contributing to MDSC generation, including Monocyte Chemoattractive Protein-1, interleukin-6, interleukin-8, and Macrophage Migration Inhibitory Factor (MIF). Of these, Macrophage Migration Inhibitory Factor is a particularly attractive therapeutic target as sulforaphane, a naturally occurring MIF inhibitor derived from broccoli sprouts, has excellent oral bioavailability. Sulforaphane inhibits the transformation of normal monocytes to MDSCs by glioma-conditioned media *in vitro* at pharmacologically relevant concentrations that are non-toxic to normal leukocytes. This is associated with a corresponding increase in mature dendritic cells. Interestingly, sulforaphane treatment had similar pro-inflammatory effects on normal monocytes in fresh media but specifically increased immature dendritic cells. Thus, we have used a simple *in vitro* model system to identify a novel contributor to glioblastoma immunosuppression for which a natural inhibitor exists that increases mature dendritic cell development at the expense of myeloid-derived suppressor cells when normal monocytes are exposed to glioma conditioned media.

## Introduction

Glioblastoma (GBM) is a devastating disease with mean survival of 14 months despite optimal therapy.[1] Immunotherapies have emerged as promising therapeutic strategies for GBM.[2] Preclinical GBM immunotherapy studies have shown excellent results[3], but human clinical trials results have been more modest.[4] Local and systemic GBM-induced immunosuppression is a significant barrier to immunotherapy.[5] Systemic immunosuppression in GBM patients reflects accumulations of immunosuppressive leukocytes such as myeloid-derived suppressor cells (MDSCs) and regulatory T-cells (Tregs) which inhibit the proliferation and activation of T cells.[6, 7] MDSCs are derived from monocytes and include both monocytic and granulocytic variants.[8] In mice, they can be defined as CD11b^+^/Gr-1^+/^Ly6C^+^ (monocytic) or CD11b^+^/Gr-1^+^/Ly6G^+^ cells.[3] In humans, accepted MDSC surface marker profiles have evolved over the past decade. Monocytic MDSCs are currently best defined as CD11b+/CD14^+^/CD15-/HLA-DR^-^ cells while granulocytic MDSCs are CD11b^+^/CD14^-^/CD15^+^ cells[9]. However, CD14+ cells almost universally also express CD11b and the necessity for excluding CD15+ cells from within monocytic MDSC definitions has only been widely accepted more recently. As result, many authors have relied only on CD14 and HLA-DR staining to identify monocytic MDSCs in cancer patients, either alone or as a surrogate after first identifying a population of MDSCs that are CD11b+/CD14+/CD15-/HLA-DR-. [10–12] Normal cells with similar surface marker phenotypes but without immunosuppressive function can occur. Therefore, true definition of MDSCs requires the demonstration of a functional ability to inhibit T cell proliferation in addition to surface marker profiling.

MDSCs are present at low baseline levels in non-cancer patients with roles in preventing autoimmune states and moderating inflammatory reactions.[13, 14] Malignant tumors subvert this natural role of MDSCs in order to protect themselves from tumor immunosurveillance.[15] Previously, we and others have shown that normal human monocytes co-cultured with GBM cells transform into both monocytic MDSCs (mMDSCs) and granulocytic MDSCs (gMDSCs).[16, 17] This same phenomenon is seen *in vivo* when syngeneic mouse monocytes are co-injected intracranially with GL261 murine glioma cells into C57BL/6 mice. The presence of increased monocytes in the mouse tumor environment from the time of implantation leads to increased tumor growth and increased intra-tumoral and systemic immunosuppressive MDSCs.[3]

The mechanisms underlying MDSC accumulation in cancers such as GBM are not clear. GBM cells are known to secrete multiple immunomodulatory cytokines into the tumor microenvironment [16–19] though these are not generally increased in patients’ serum. Monocytes are continually trafficking in and out of the tumor microenvironment.[20] During this time they may undergo immunoeducation leading to their transformation into MDSCs.[3, 21] This process could occur secondary to cell-cell contact between naïve monocytes and GBM cells or, alternatively, due to exposure to the cytokine-rich intra-tumoral environment.[3, 16] Findings in our *in vivo* glioma model suggest these MDSCs then re-enter the systemic circulation [3] where they inhibit T-cells proliferation and induce apoptosis in activated T-cells.

Blocking the transformation of normal monocytes into MDSCs could have major implications in immunotherapy. A non-immunosuppressed GBM or cancer patient may be able to mount a more robust anti-tumor response spontaneously or in response to a vaccine. However, studies of human MDSC biology in cancer have been hampered by both limited availability of patient material and cumbersome monocyte / cancer cell co-culture systems. Therefore, in this study we aimed to create a cell free human *in vitro* MDSC model and screen this model for targetable molecules contributing to MDSC development.

## Methods

### Glioblastoma cell culture

Fresh human glioblastoma tumor tissue was obtained at surgery. Human specimens for this research were obtained with written, informed consent after approval of this project by the Mayo Clinic Institutional Review Board (Mayo Clinic IRB#12–003458). Single cell suspensions were generated by cutting tissue into small pieces followed by repeated aspiration through an 18-gauge needle. Cell cultures were originally established as brain tumor stem cell neurosphere lines in minimally hormonally supplemented serum-free media containing EGF and FGF as previously described.[22] They were subsequently transferred to DMEM with 1% penicillin/streptomycin and 10% fetal calf serum (FCS) to generate the differentiated human GBM cell lines used in this study. These differentiated cultures were grown in a monolayer in T75 tissue culture flasks at 37°C, 5% CO_2_.

### Human monocyte isolation

Anonymized, discarded leukoreduction chambers were obtained from normal, healthy blood donors to the Mayo Clinic Blood Bank.[23] The chambers were flushed through with 30 mL of red cell lysis buffer into a sterile 50 mL tube. The tubes were vortexed and centrifuged at 1300 rpm for 4 minutes. The supernatant was aspirated leaving a pellet with residual interspersed red blood cells. This pellet was resuspended in 30 mL of red cell lysis buffer and centrifuged again at 1300 rpm for 4 minutes. The supernatant was aspirated, leaving a leukocyte pellet which was washed with sterile PBS and centrifuged again with the same settings. The pellets were then resuspended in the recommended amount of MACS buffer and CD14 magnetic beads (Miltenyi Biotec, San Diego, CA). The cell-bead mixture was incubated for 15 minutes at 4°C. The CD14^+^ cells were then collected using Millitenyi LS columns (Miltenyi Biotec, San Diego, CA) on a magnetic assisted cell sorting device.

### In vitro MDSC assay

Three different human glioblastoma cell lines (BT114, BT116, and BT120) were seeded into 6-well plates at a density of 100,000 cells per well. The cells were cultured for 24 hours in DMEM with 10% FBS and 1% pen/strep under normoxic conditions (room air plus 5% CO_2_). The supernatant was discarded and replaced with DMEM containing only 1% pen/strep, and cultured for 48 hours under normoxic conditions. This glioma conditioned media (GCM) was aspirated and centrifuged at 1300 rpm for 5 minutes to remove cells. The supernatant was then aspirated and frozen at -20°C for future use. Healthy donor monocytes were isolated and seeded at a density of 3 million cells per well in a 12-well plate in 2 ml of each of the three different glioblastoma conditioned medias (GCM)s and in unconditioned DMEM and 1% pen/strep. The cells were then placed in either a hypoxic environment (1% O_2_) or normoxic environment (21% O_2_) and incubated for 48 hours. Monocytes were aspirated and washed in FACS buffer. Cells were stained in 100 μL FACS buffer with 2 μL each of CD14-FITC, CD11b-PE, CD15-PerCp, and HLA-DR-v450 antibody or CD14-FITC conjugated antibody and HLA-DR PerCP or PE conjugated antibodies (eBioscience). Stained cells were analyzed by flow cytometry using FlowJo Data Analysis Software (FlowJo LLC Ashland, OR). Forward and side scatter gating was used to exclude residual lymphocytes and debris. CD11b / CD14 / CD15 / HLA-DR expression patterns in this cell population was then determined.

### Lyophilization of glioma conditioned media (GCM)

Glioma conditioned media was transferred to 50 mL Falcon tubes and placed under refrigerated low pressure to create a lyophilized powder. The powder was stored at -20°C and then reconstituted in sterile distilled water with the same volume as the original pre-lyophilization sample.

### Cell staining and confocal imaging

Glioma-conditioned monocytes harvested from the MDSC assay were stained in the same manner as for flow cytometry and fixed with 5% formalin. The cells were dropped onto a glass slide and mounted with Prolong-Gold (Invitrogen, Carlsbad, CA) mounting medium containing DAPI. The cells were then viewed with a Zeiss LSM confocal microscope.

### Cytokine array

Cytokine analysis using the Human Cytokine Array G5 Microarray from Raybiotech (Norcross, GA) was carried out per the manufacturer’s instructions in control unconditioned DMEM (Life Technologies, Carlsbad, CA) and glioma-conditioned media from the three human glioblastoma cell cultures used to generate GCM in the MDSC experiments. The full list of the 80 measured cytokines is shown in Table 1 and at http://www.raybiotech.com/human-cytokine-array-g5-4.html. Samples were stored at -80°C, thawed on ice when ready to be used, and centrifuged for 30 minutes at 14,000 rpm at 4°C to remove any debris. Media (100 μL per sample) was loaded into the array and incubated at 4°C overnight. The intensities of signals of the conjugated Cy3 fluor (555 nm) were quantified by densitometry using GenePix 4000B scanner (Molecular Devices, Sunnyvale, CA) and analyzed GenePix Pro 6.1 software (Molecular Devices, Sunnyvale, CA). The results were normalized to unconditioned media using the internal positive controls provided on each array. The normalized DMEM sample value for each cytokine was then use as a zero value and the respective sample values were adjusted accordingly. Readings less than zero were recorded as zero. A cut-off of the mean + 2 standard deviations of the negative controls per array was used to assess the threshold of a true positive signal.

**Table 1:** Expression of biomarkers in glioma conditioned media.

Name	DMEM	114 GCM	116 GCM	120 GCM
**Angiogenin**	**0**	**2676**	**3454**	**2668**
**BDNF**	**0**	**0**	**0**	**131**
**Eotaxin-3**	**0**	**0**	**0**	**0**
**Flt-3 Ligand**	**0**	**0**	**3**	**50**
**G-CSF**	**0**	**0**	**0**	**0**
**GDNF**	**0**	**0**	**0**	**0**
**GM-CSF**	**0**	**0**	**0**	**155**
**GRO**	**0**	**0**	**111**	**749**
**GRO-alpha**	**0**	**0**	**0**	**0**
**HGF**	**0**	**0**	**1026**	**0**
**IFN-gamma**	**0**	**0**	**0**	**0**
**IGF-I**	**0**	**0**	**0**	**7**
**IGFBP-1**	**0**	**0**	**0**	**109**
**IGFBP-2**	**0**	**398**	**1062**	**636**
**IGFBP-3**	**0**	**0**	**559**	**152**
**IL-1 alpha**	**0**	**0**	**0**	**0**
**IL-1 beta**	**0**	**0**	**0**	**122**
**IL-10**	**0**	**0**	**0**	**0**
**IL-12 p70**	**0**	**0**	**0**	**0**
**IL-13**	**0**	**0**	**0**	**0**
**IL-15**	**0**	**0**	**0**	**0**
**IL-2**	**0**	**0**	**0**	**0**
**IL-3**	**0**	**0**	**0**	**0**
**IL-5**	**0**	**0**	**0**	**0**
**IL-6**	**0**	**178**	**2157**	**9665**
**IL-7**	**0**	**0**	**0**	**0**
**IL-8**	**0**	**103**	**5303**	**15294**
**IP-10**	**0**	**5**	**0**	**0**
**LIF**	**0**	**0**	**0**	**0**
**MCP-1**	**0**	**2993**	**40301**	**31031**
**MCP-2**	**0**	**0**	**0**	**0**
**MCSF**	**0**	**0**	**0**	**0**
**MIF**	**0**	**1932**	**4254**	**9857**
**MIG**	**0**	**0**	**0**	**0**
**MIP-3 alpha**	**0**	**0**	**0**	**88**
**Oncostatin M**	**0**	**0**	**0**	**0**
**Osteopontin**	**0**	**0**	**1851**	**225**
**Osteoprotegerin**	**0**	**0**	**0**	**560**
**PARC**	**0**	**0**	**0**	**0**
**PDGF-BB**	**0**	**0**	**0**	**86**
**PIGF**	**0**	**0**	**140**	**0**
**RANTES**	**0**	**0**	**0**	**319**
**SCF**	**0**	**99**	**0**	**91**
**SDF-1**	**0**	**0**	**0**	**0**
**TGF-beta 1**	**0**	**0**	**0**	**7**
**TGF-beta 2**	**0**	**0**	**0**	**0**
**TIMP-1**	**0**	**601**	**2539**	**1982**
**TIMP-2**	**0**	**8430**	**6869**	**8951**
**TNF-alpha**	**0**	**0**	**0**	**0**
**TNF-beta**	**0**	**0**	**0**	**0**
**VEGF**	**0**	**0**	**781**	**636**

**Cytokine expression in GCM**. A cytokine array demonstrates a panel of 80 cytokines from fresh media and glioma-conditioned media derived from three human glioma cell lines (BT114, BT116, BT120). Numbers represent mean fluorescent units. All numbers were zeroed to the fresh media which served as a control. Red fields represent values greater than 2 standard deviations above the negative standard mean. Green fields represent non-significant positive values. Note: these cell lines are all IDH-wild type but have not been extensively characterized genetically otherwise.

### Determining MIF concentration in GCM

MIF concentration in three separate biological replicates of glioma conditioned media from BT114, BT116, and BT120 were prepared as outline above. MIF concentration was then determined by enzyme linked immunosorbant assay (ELISA) using a commercially available kit (Human MIF Quantikine ELISA; R&D Systems, Minneapolis, MN) per the manufacturer’s instructions.

### Assessing effects of MIF in GCM on MDSC generation from monocytes

CD 14 monocytes were isolated from anonymized donor leukocytes as before. Cells were plated (1.5x10^6^ cells / mL) in 96 well plates in a total of 200 ul. Four treatment groups were assessed: monocytes in DMEM with 1% Penicillin/Streptomycin, BT 116 GCM, BT 116 GCM + 20 ug/ml MIF neutralizing antibody (Millipore, Billerica, MA), and BT 116 GCM + 40 ug/ml MIF neutralizing antibody. Cells were incubated under hypoxic conditions (1% O_2_) for 48 hours at 37°C, stained using CD14-FITC and HLA-DR-PerCP, and analyzed by flow cytometry. Two separate experiments (each conducted in triplicate) were performed. Similar experiments and replicates were performed with different monocyte donors using DMEM with 1% Penicillin/Streptomycin +/- 50 ng/ml or 100 ng/ml recombinant human MIF protein (R&D Systems, Minneapolis, MN) [24].

### MIF enzymatic assay

L-Dopachrome methyl ester was prepared by adapting a protocol from Healy et al.[25] A total of 19 ml of reaction buffer (1mmol/L EDTA and 500μmol/L Bis-Tris in distilled H20) was prepared. Sodium periodate (500 μl of 24mmol/L) and L-Dopa (L-3,4-Dihydroxyphenylalanine methyl ester hydrochloride) (500 μl of 1μmol) were added to the buffer. The substrate was incubated for 5 minutes at 25°C in the dark with a final pH of 6.3. All reagents were purchase from Sigma-Aldrich (Saint Louis, MO). Dopachrome was synthesized immediately before use. Glioma conditioned media was created from glioma cells lines: BT 114, BT 116, and BT 120, as described above. DMEM or GCM (100 μL/well) was transferred into flat bottom 96 well plates. R-Sulforaphane (SFN) (LKT laboratories, St Paul, MN) was added to the treated wells with a final SFN concentration of either 5 μM or 10 μM. The plate was immediately placed in a plate reader and read at 475 nm absorbance for 40 cycles.

### Cell death assay

Lymphocytes and monocytes were isolated from peripheral blood mononuclear cells from three healthy donors by magnetic bead sorting using CD3 and CD14 conjugated antibodies (Miltenyi Biotec, Sand Diego, CA) respectively as described above. Lymphocytes, monocytes, or human glioma cells (BT114, BT116, BT120) were transferred to 12 well plates (3 million cells/well) with 2 ml of DMEM + 10% FCS and 1% penicillin streptomycin. Sulforaphane was added to each well to create final concentrations of 200 uM, 100 uM, 50 uM, 20 uM, or 10 uM. Cells were cultured for 48 hours at 37°C and 5% CO2. Leukocytes were harvested by aspiration, placed in 5 ml Falcon glass tubes, and centrifuged (1200 rpm for 4 minutes). The media was aspirated and the pellet was resuspended in 100 μL of MACS buffer with 2 μL of Annexin V-FITC and 7AAD (7-aminoactinomycin D). The cells were analyzed by flow cytometry.

### Blocking MDSC formation with SFN

MDSCs were generated using GCM as described above. Monocytes (3 x 10^6^ / well) were loaded in each well of a 12 well plate with in 2ml of either serum-free DMEM, serum-free GCM, serum-free GCM plus SFN (final concentration 5μM), and serum free GCM plus SFN (final concentration 10 μM). GCM from BT114, BT116, and BT120 was used. The monocytes were incubated at 37°C for 48 hours in hypoxic conditions (1% O2). The monocytes were then stained and analyzed in a flow cytometer for CD14 and HLA-DR status. This was repeated with monocytes from three unique donors.

### T-cell proliferation assay

Glioma-conditioned media using BT116 cell line was created using the protocol above. Autologous human CD14^+^ monocytes and CD3^+^ lymphocytes were isolated using magnetic assisted cell sorting. The CD14+ monocytes were then cultured in the DMEM + 1% penicillin/streptomycin, DMEM + 1% penicillin/streptomycin + 5μM SFN, DMEM + 1% penicillin/streptomycin + 10μM SFN, BT116 GCM, BT116 GCM + 1% penicillin/streptomycin + 5μM SFN, and BT116 GCM + 1% penicillin/streptomycin + 10μM SFN for 48 hours under hypoxic conditions (1% O_2_). The monocytes were collected and a portion was analyzed for CD14 and HLA-DR by FACS analysis to confirm the development or blocked development of MDSCs. CD3^+^ T-cells were stained with carboxyfluorescein succinimidyl ester (CFSE) at 5μM in room temperature phosphate buffered saline (PBS) for 15 minutes. The samples were then centrifuged and washed with PBS, resuspended in DMEM with 10% FCS, and 1% penicillin/streptomycin, and seeded into a 96-well plate at a density of 8 x 10^4^ cells per well with 8 x 10^4^ anti-CD3/CD28 Dynabeads per well (Life Technologies, Carlsbad, CA). The previously harvested, conditioned, and treated monocytes were then washed with PBS to remove any residual SFN, and seeded in to the same wells with the T-cell at a ratio of 1:1. The 96-well plate was cultured for 5 days under normoxic conditions. The cell mixtures were removed from the 96-well plates and placed in 1.5 mL eppendorf tubes. The magnetic beads were removed by placing each sample on a magnet for one minute and harvesting the supernatant. The cells were resuspended in FACS buffer and analyzed for CFSE signal degradation in a flow cytometer. This experiment was repeated three times with unique leukocyte donors.

### Determination of PD-L1 expression in monocytes treated with sulforaphane

CD14^+^ monocytes were isolated from three unique anonymized healthy donor leukocytes as described earlier. Cells were suspended in 2ml of media at a density of 1.5x10^6^ cells per milliliter in 12 well plates. Four treatment groups were created: CD14^+^ monocytes in DMEM with 1% Penicillin/Streptomycin, GCM, GCM + 5μM SFN, and GCM + 10μM SFN. The plates were then incubated for 48 hours at 37°C under hypoxic conditions (1% O_2_). The cells were then washed and stain as described earlier with CD 14-FITC, HLA-DR-PerCP, and PD-L1-APC. Stained cells were analyzed by flow cytometry.

### Statistical analysis

Statistical analysis was performed with GraphPad Prism software. Two-tailed Student T tests or two-way analysis of variance (ANOVA) were performed for single or multiple groups comparisons, respectively. Tukey’s analysis was used when appropriate.

## Results

### Normal human monocytes cultured in glioma-conditioned media under hypoxic conditions are enriched for MDSCs

**Fig 1:**
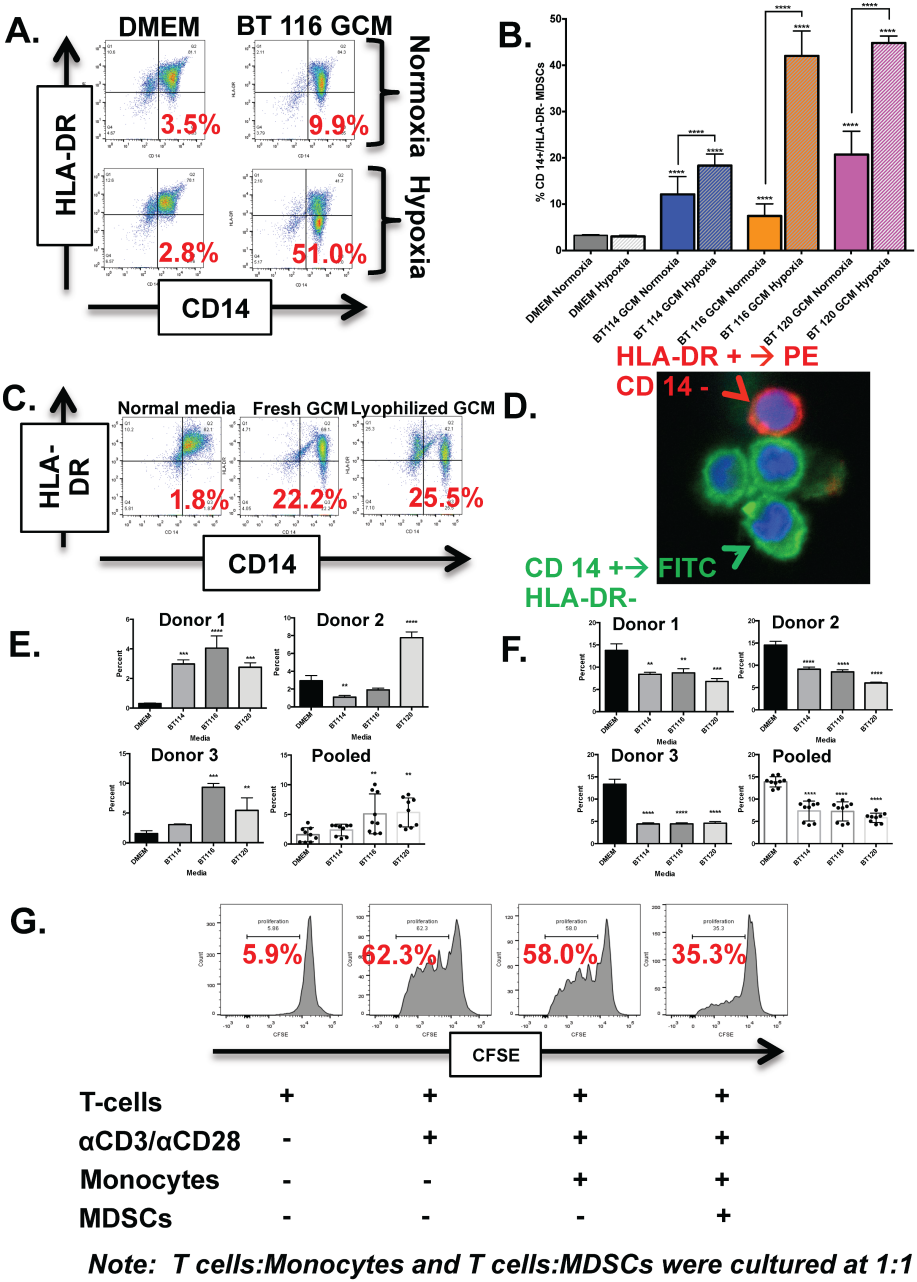
Monocytes cultured in glioma-conditioned media (GCM) under hypoxic conditions become CD14+/HLA-DR- mMDSCs. A) Representative dots plots showing CD 14 and HLA-DR expression by healthy monocytes cultured in normoxic and hypoxic serum free media (top) versus monocytes cultured in normoxic and hypoxic GCM (bottom). Monocytes cultured in hypoxic GCM have the highest concentration of CD 14+/HLA-DR- mMDSCs (percent shown in red). B) Bar graphs showing significant increases in mMDSC formation across three healthy donor monocytes in each of the three GCM lines and significant increases in MDSCs when comparing monocytes cultured in hypoxic GCM to monocytes cultured in normoxic GCM. C) Representative dot plots showing that lyophilized and reconstituted GCM is as effective as fresh GCM in generating CD 14+/HLA-DR- MDSCs. D) Confocal micrographs of normal human monocytes after hypoxic culture for 48 hours in GCM (BT116). The cells are stained with CD14 (green) and HLA-DR (red). CD14^+^/HLA-DR^-^ MDSCs (green) can be clearly distinguished from cells with more HLA-DR expression (red or mixed). E) Bar graphs showing increased monocytic MDSCs after culture in glioma-conditioned media for individual monocyte donors (n = 3) and pooled results from all donors. F) Bar graphs showing decreased granulocytic MDSCs after culture in glioma-conditioned media for individual monocyte donors (n = 3) and pooled results from all donors. G) Histograms demonstrating decreased T cell proliferation (shift to the left with CFSE staining) with anti-CD3 / anti-CD28 antibody stimulation in the presence of MDSC-enriched monocytes generated by culture in glioma-conditioned media (“MDSCs”) compared with monocytes cultured in fresh media. ** = P < 0.01, *** = P < 0.001 **** = P < 0.0001.

Monocytes normally express HLA-DR. Cells with a CD14^+^/HLA-DR^-^ phenotype are generally considered to represent monocytic MDSCs when they arise in the presence of cancer cells and have demonstrated immunosuppressive properties [10–12], though more stringent characterization as CD11b+ / CD14+ / HLA-DR- / CD15- can also be helpful [9]. Hypoxia is present within glioblastoma tumors where monocytes infiltrate, has immunological consequences, and has been associated with increased MDSC production in other systems [26–28]. We therefore sought to determine MDSC frequency in normal human monocytes cultured in fresh media or glioma-conditioned media under both normoxic and hypoxic conditions. Normal CD14^+^ monocytes cultured in fresh media under both hypoxia and normoxia are largely positive for HLA-DR as expected (Fig 1A top). However, CD14^+^ monocytes cultured in glioma-conditioned media show a marked increase in CD14^+^/HLA-DR^-^ MDSCs (Fig 1A bottom). This phenotypic change is enhanced under hypoxic (1% O_2_) culture conditions (Fig 1A bottom right). This pattern was consistent across three different human glioma-conditioned medias (BT114, BT116, BT120) and three separate healthy monocyte donors. (Fig 1B). Significantly increased CD14+/ HLA-DR- MDSCs were seen for all three monocyte donors cultured under both normoxia and hypoxia in glioma-conditioned media. For normoxic conditions, analysis was as follows: DMEM vs BT 114 GCM, p<0.05 95% CI (-9.490 to -8.160); DMEM vs BT 116 GCM, p<0.05 95% CI (-4.820 to -3.490); DMEM vs. BT 120 GCM, p<0.05 95% CI (-18.09 to -16.76). For hypoxic conditions, results were: DMEM vs BT 114 GCM, p<0.05 95% CI -15.72 to -14.40); DMEM vs BT 116 GCM, p<0.05 95% CI(-39.42 to -38.10); DMEM vs. BT 120 GCM, p<0.05 95% CI(-42.22 to -40.90). Normoxic conditions had significantly reduced CD14+/HLA-DR- MDSC number compared to hypoxic conditions: normoxic BT114 GCM vs. hypoxic BT114 GCM, p <0.05, 95% CI(-6.900 to -5.570); normoxic BT116 GCM vs. hypoxic BT116 GCM, p<0.05, 95% CI (-35.27 to -33.94); normoxic BT120 GCM vs. hypoxic BT120 GCM, p<0.05, 95% CI(-24.80 to -23.47).

### Normal human monocytes cultured in lyophilized and reconstituted GCM become MDSCs

It would be logistically simpler to generate MDSCs from normal monocytes with glioma-conditioned media if this media could be prepared in advance and stored rather than prepared fresh for each experiment. We therefore sought to determine if lyophilized glioma-conditioned media retained its ability to induce MDSCs. Both fresh and lyophilized GCM were robust in generating CD14^+^/ HLA-DR^-^ MDSCs (Fig 1C).

### MDSCs generated in-vitro can be observed with confocal microscopy

We were conscious that the CD14^+^/HLA-DR^-^ population seen by flow cytometry in normal monocytes exposed to GCM did not always form a distinct cluster in the FACS dot plots but instead appeared to be a relative reduction in HLA-DR expression in the same population of cells that were strongly CD14^+^/HLA-DR^+^ after culture in fresh media. To help confirm that this relative reduction in HLA-DR expression was not simply artifact, we additionally analyzed these cells by confocal microscopy. MDSCs generated with BT116-conditioned media were stained with FITC conjugated CD14 antibody and PE conjugated HLA-DR antibody and assessed by confocal microscopy. Fig 1D shows an obvious differentiation between CD14^+^/HLA-DR^-^ MDSCs, and CD14^-^/HLA-DR^+^ monocytes.

### MDSCs generated in vitro can be further classified by CD11b and CD15 expression

Currently accepted best surface marker profiles are CD11b+ / CD14+ / CD15- / HLA-DR- cells for human monocytic MDSCs and CD11b+ / CD14- / CD15+ cells for granulocytic MDSCs [9]. We cultured healthy donor monocytes in hypoxic conditions in either serum-free fresh media or glioma-conditioned media and analyzed by flow cytometry for CD11b, CD14, CD15, and HLA-DR expression patterns. S1 Fig shows representative histograms and dot plots demonstrating our gating strategy. For both serum-free media and glioma conditioned media, the majority of CD11b+ / CD14+ / HLA-DR- cells were also CD15- (i.e. monocytic MDSCs) but only a minority of CD11b+ / CD14- cells were also CD15+ (i.e. granulocytic MDSCs), The frequency of monocytic MDSCs as a percentage of CD11b+ cells generally increased following exposure to three different glioma-conditioned medias for three different monocyte donors (Fig 1E), though this was most marked for BT116 and BT120 conditioned media. Interestingly, the frequency of granulocytic MDSCs as a percentage of CD11b+ cells uniformly decreased after exposure to glioma-conditioned media (Fig 1F).

### MDSCs generated in vitro inhibit T cell proliferation

Simply having surface marker profiles compatible with MDSCs (e.g. CD14^=^/HLA-DR^-^) is not sufficient to identify cells as myeloid-derived suppressor cells. The cells in question must also be functionally immunosuppressive. We therefore tested the ability of our *in vitro*-generated “MDSCs” to inhibit T cell proliferation. T cells (CD3+) cultured alone or in the presence of anti-CD3 / anti-CD28 antibodies to stimulate proliferation. Comparison was made to T cells stimulated by anti-CD3/anti-CD28 antibodies in the presence of either autologous monocytes or autologous MDSC-enriched monocytes (“MDSCs”) generated by culture with glioma-conditioned media. Proliferation was determined by CFSE staining and flow cytometry. Fig 1G shows clear inhibition of T cell proliferation in the presence of MDSCs but not normal monocytes.

### Cytokines present in glioma conditioned media

To identify candidate factors within glioma-conditioned media contributing to MDSC development, GCM was analyzed using a Ray Biotech cytokine array. Fresh DMEM was used as a negative control. Cytokine levels relative to DMEM are shown as a relative fold increase or decrease (Table 1). Significant increases were seen for multiple cytokines, including angiogenin, Insulin-like growth factor binding protein-2 (IGFBP-2), interleukin-6 (IL-6), interleukin-8 (IL-8), monocyte chemoattractive protein-1 (MCP-1/CCL2), macrophage migration inhibitory factor (MIF), tissue inhibitor of metalloproteinase-1 (TIMP-1), and TIMP-2. Of these, MIF was of particular interest as it has been implicated in MDSC development previously but its role in glioma-induced MDSC accumulation is unknown.

### Neutralizing MIF eliminates GCM mediated CD14+/HLA-DR- MDSC formation

**Fig 2:**
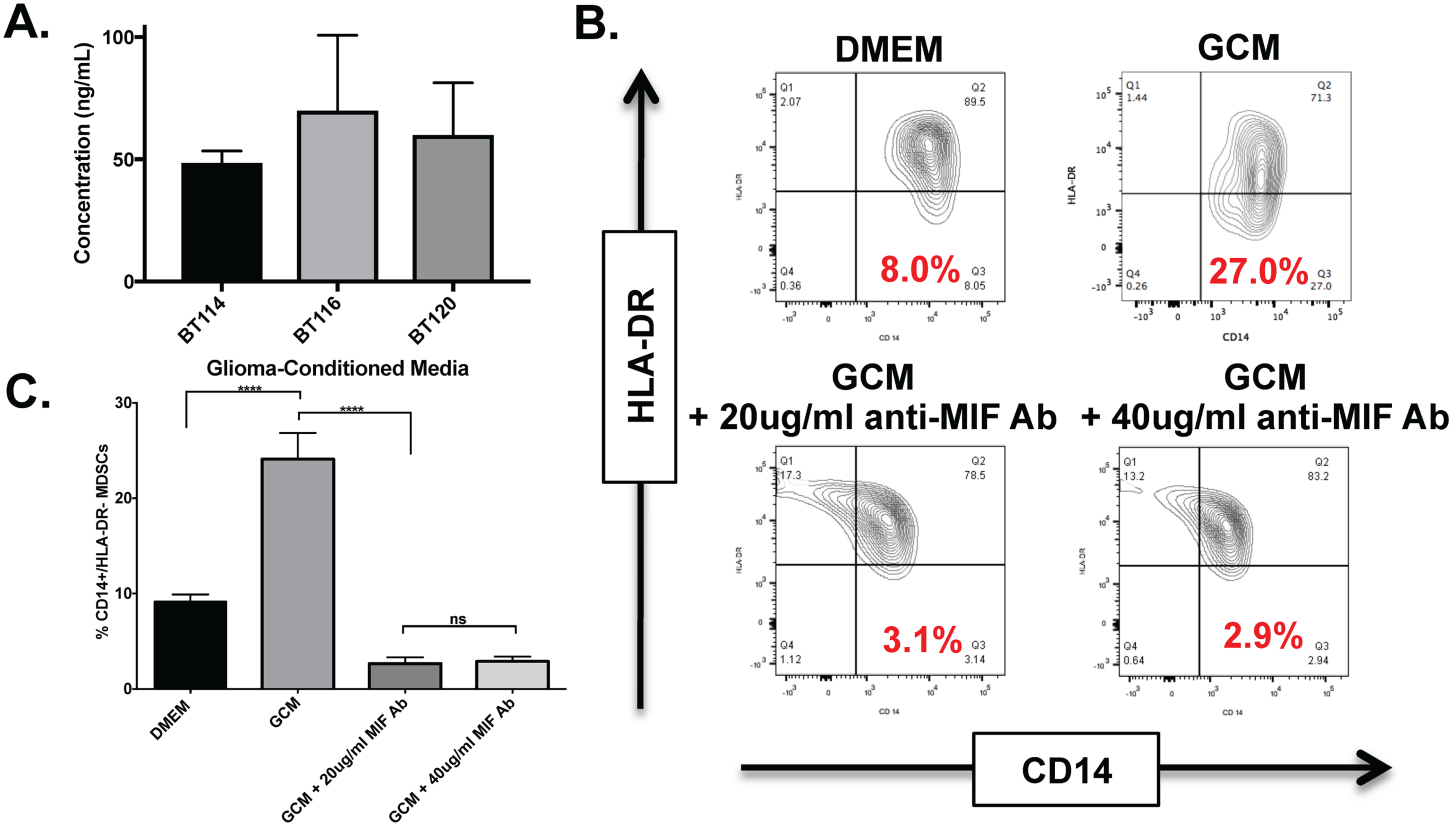
Macrophage Migration Inhibitory Factor (MIF) is a critical component of GCM promoting MDSC formation. A) ELISA results confirming the presence of MIF in glioma-conditioned media. An MIF neutralizing antibody blocks the formation of CD14+/HLA-DR- cells when cultured in hypoxic (1% O_2_) GCM. B) Representative dot plots showing CD 14 and HLA-DR expression in monocytes in serum free DMEM (top left), GCM from BT 116 (top right), GCM + 20 ug/ml anti-MIF Ab (bottom left), and GCM + 40 ug/ml anti-MIF Ab (bottom right). There is a reduction in the amount of CD14+/HLA-DR− cells (mMDSC’s) in the anti-MIF treated monocytes (percent shown in red). C) Bar graphs showing a significant reduction in the mean frequency of CD14+/HLA-DR- cells generated from monocytes cultured in GCM in the presence of anti-MIF antibodies. **** = P < 0.0001.

To confirm the presence of MIF in our glioma-conditioned media, we performed an ELISA with three separate biological replicates from our three glioma lines (BT114, BT116, BT120). This demonstrated MIF concentrations of between 48.6 ng/mL to 69.9 ng/mL, although there were no significant differences between different GCM’s (Fig 2A). S2 Fig shows the MIF ELISA standard curve obtained with the kit. Note that all glioma-conditioned media samples required a 1:10 dilution with PBS to bring them within the range of this standard curve. The data in Fig 2A has been adjusted for this dilution factor. Healthy donor monocytes (n = 2) were cultured in BT116 GCM with or without 20 ug/ml or 40 ug/ml MIF neutralizing antibody (MABF111, EMD Millipore, Billerica, MA)[29] under hypoxic (1% O_2_) conditions as described above. There was a significant increase in CD14+/HLA-DR- MDSCs arising from CD14^+^ monocytes cultured in GCM compared to monocytes cultured in DMEM, as expected (p<0.0001, 95% CI -17.20 to -12.71). (Fig 2B and 2C). This was significantly and markedly decreased if monocytes were cultured in GCM in the presence of MIF neutralizing antibody compared to GCM alone (20 mg/ml MIF neutralizing antibody: p<0.0001, 95% CI 19.19 to 23.67; 40 mg/ml MIF neutralizing antibody: p<0.001, 95% CI 18.95 to 23.44) and compared to DMEM alone (20 mg/ml MIF neutralizing antibody: p = 0.0002, 95% CI 4.234 to 8.721; 40 mg/ml MIF neutralizing antibody: p = 0.003, 95% CI 4.002 to 8.488). Of note, we also cultured healthy monocytes in fresh media with or without the addition of exogenous MIF (R&D Systems). This did not increase the frequency of CD14+ / HLA-DR- MDSCs (data not shown), suggesting that while MIF is necessary for MDSC induction it is not sufficient but works in concert with other factors contained within GCM.

### MIF tautomerase conversion of L-Dopachrome to DHIC is inhibited by sulforaphane

**Fig 3:**
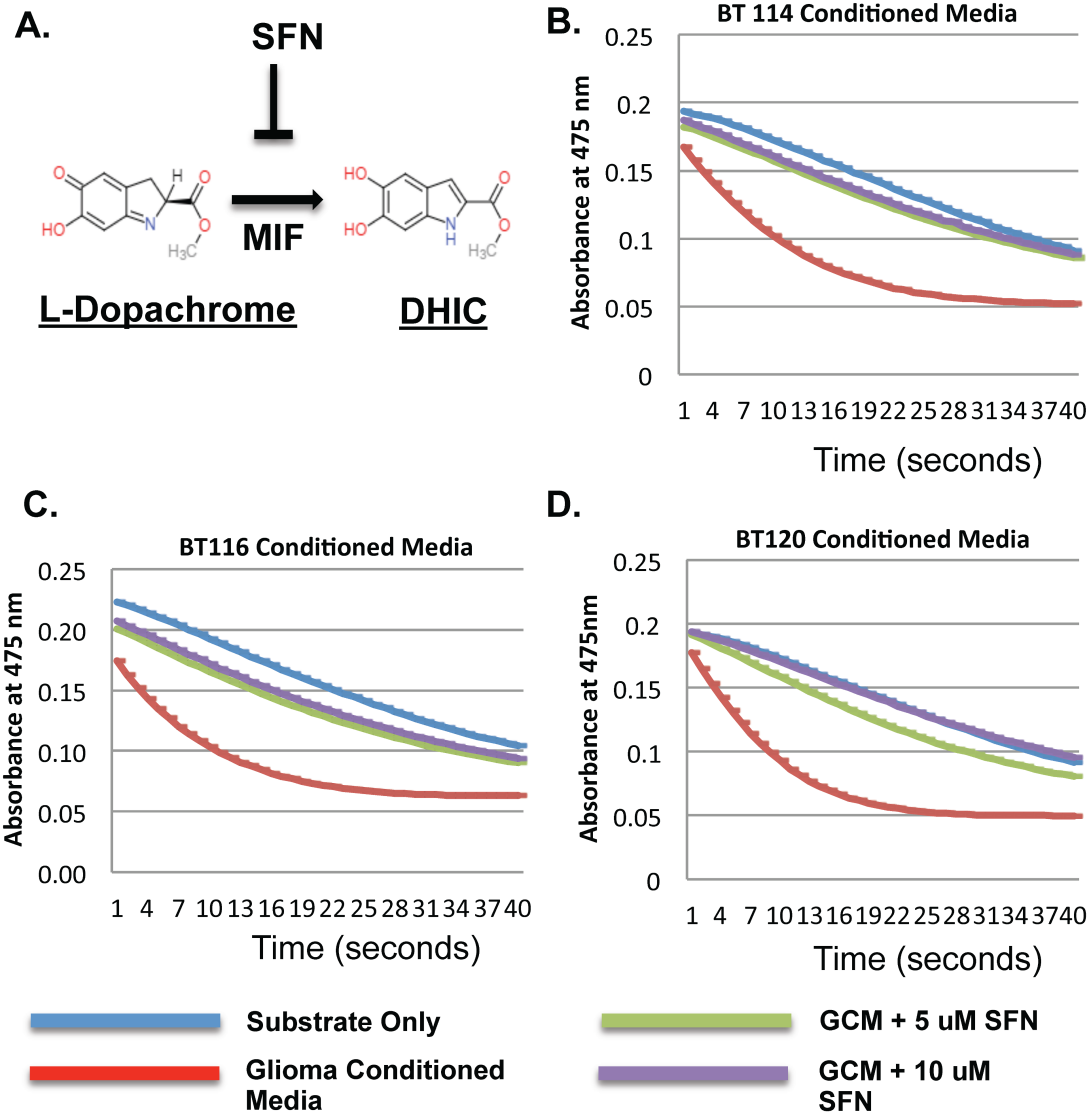
Sulforaphane (SFN) inhibits MIF’s keto-enol tautomerase activity. A) Schematic showing SFN inhibiting MIF-mediated L-Dopachrome (methyl ester of 2-carboxy-2,3-dihydroxyindole-5,6-quinone) conversion to DHIC (methyl ester of 5,6-dihydroxyindole-carboxylate). B) Graph showing the effect of BT114 GCM and SFN on L-Dopachrome conversion to DHIC. L-Dopachrome is measured by its absorbance at 475 nm and degrades spontaneously in solution in a linear fashion over time (x axis = time where each integer equals 50 seconds). Addition of BT114 GCM causes L-Dopachrome to degrade exponentially. This reverts back to linear (i.e. spontaneous) levels with addition of SFN. C) Graph showing similar effects of SFN on BT116 GCM-mediated L-Dopachrome degradation to DHIC. D) Graph showing similar effects of SFN on BT120 GCM-mediated L-Dopachrome degredation to DHIC. For all three GCM’s (BT114, BT116, BT120) statistically different slopes (P < 0.001) were seen over the first 5 minutes of the reaction when comparing GCM + 5uM SFN or GCM + 10 uM SFN to GCM alone.

Sulforaphane (SFN) is a naturally occurring and orally available compound found in broccoli sprouts that is thought to have anti-MIF properties. We wished to see if this inhibited MIF found within GCM. MIF has keto-enol tautomerase activity capable of converting L-Dopachrome to 5,6-dihydrooxyindole-2-carboxylic acid (DHIC). (Fig 3A). L-Dopachrome is orange in color and can be measured by absorbance at 475 nm but its metabolite, DHIC, is colorless. Thus, conversion of L-Dopachrome to DHIC can be measured by decreasing absorbance at 475 nm [30]. L- Dopachrome was prepared fresh in the lab as described in the methods and used promptly as it non-enzymatically converts in a linear fashion over time to DHI (5,6-dihydroxyindole).[31] All three GCMs demonstrated enzymatic reduction of L-Dopachrome (exponential) compared to the baseline non-enzymatic linear rate of reduction. When sulfopraphane was added for a final concentration of 5 uM or 10 uM, a dose dependent inhibition of L-Dopachrome reduction was seen. (Fig 3B–3D). To compare the rate of reduction of L-Dopachrome we compared the slopes over the first 5 minutes. GCM to compared to GCM + 5 uM SFN and GCM + 10 uM SFN had significantly reduced rates of reduction (P < 0.001) for all three glioma-conditioned medias.

### Sulforaphane is non-toxic to leukocytes at concentrations that inhibit MIF

**Fig 4:**
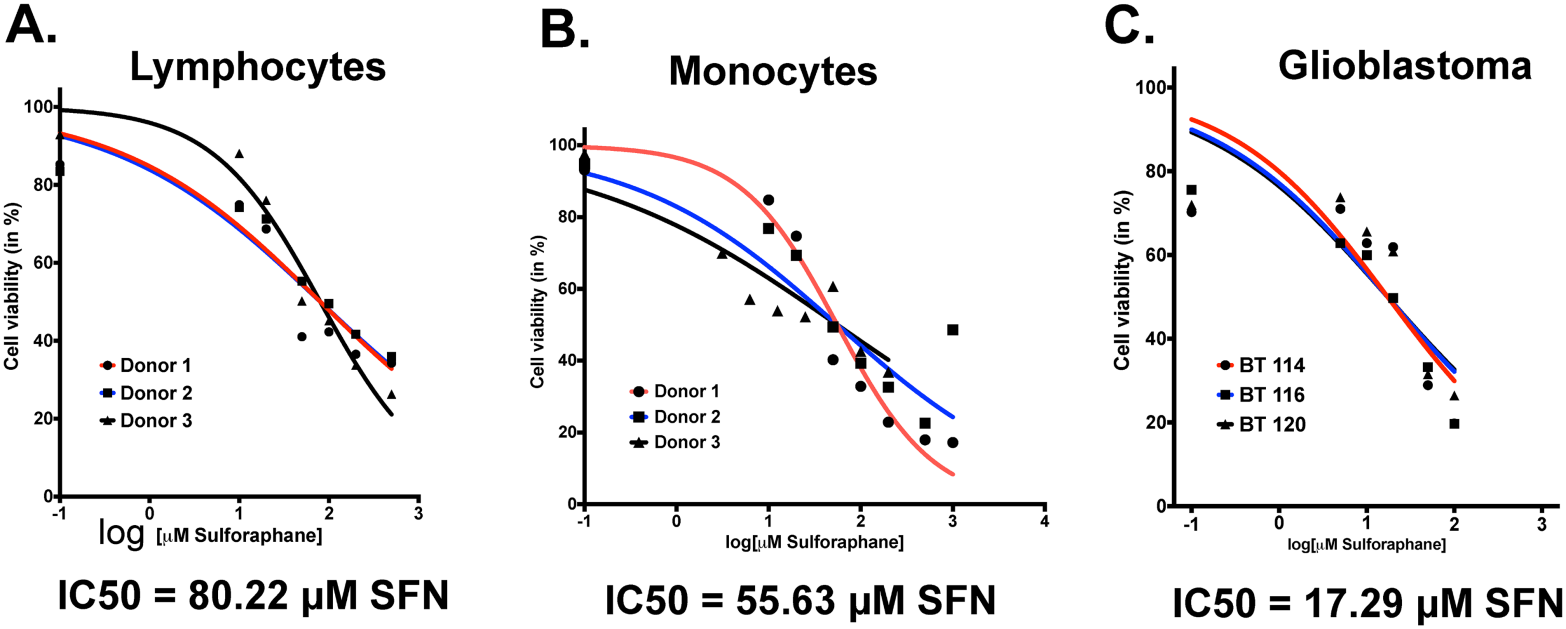
SFN is non-toxic to leukocytes but toxic to glioblastoma cells at low/moderate concentration. A) As measured by 7-AAD staining, the mean IC50 of SFN on CD3+ lymphocytes obtained from 3 different donors was 80.22 uM (p = 0.08, 95% CI (67.68–95.09)). B) The IC50 of SFN on CD14+ monocytes obtained from 3 different donors was 55.63 uM (p = 0.34, 95% CI(42.27–73.21)). C) The IC50 of SFN on GBM cells from 3 different cell lines (BT114, BT116, and BT120) was lower at 21.59 uM (p = 0.08, 95% CI(17.48–26.67)). Note that there are no statistically significant differences in IC50’s for a given cell type (lymphocyte, monocyte, GBM cell) across different donors.

We wished to determine if concentrations of sulforaphane that inhibit MIF enzymatic function *in vitro* (5–10 μM) had obvious toxicity to normal leukocytes or glioblastoma cells. Lymphocytes from three healthy donors cultured in increasing sulforaphane concentrations for 48 hours demonstrated an IC50 of 80.22 μM SFN (p = 0.08, 95% CI (67.68–95.09)) (Fig 4A) while monocytes had an IC50 of 55.63 μM SFN (p = 0.34, 95% CI(42.27–73.21)) (Fig 4B). This suggests leukocyte toxicity only at much higher concentrations than those required to inhibit MIF. Interestingly, three human GBM lines (BT114, BT116, BT120) had a lower IC50 of 21.59 μM (p = 0.08, 95% CI(17.48–26.67)) (Fig 4C), suggesting that GBM cells might be sensitive to SFN-mediated killing at concentrations that inhibit MIF but are not toxic to leukocytes. The p values represent the probability of the donors or cell lines having different susceptibilities to SFN compared to donors or cell lines of the same cell type. As expected, there were no significant differences in SFN susceptibility for a particular cell type (lymphocyte, monocyte, GBM cell) across different donors / sources.

### Sulforaphane reduces MDSC frequency and PD-L1 expression in monocytes exposed to GCM

**Fig 5:**
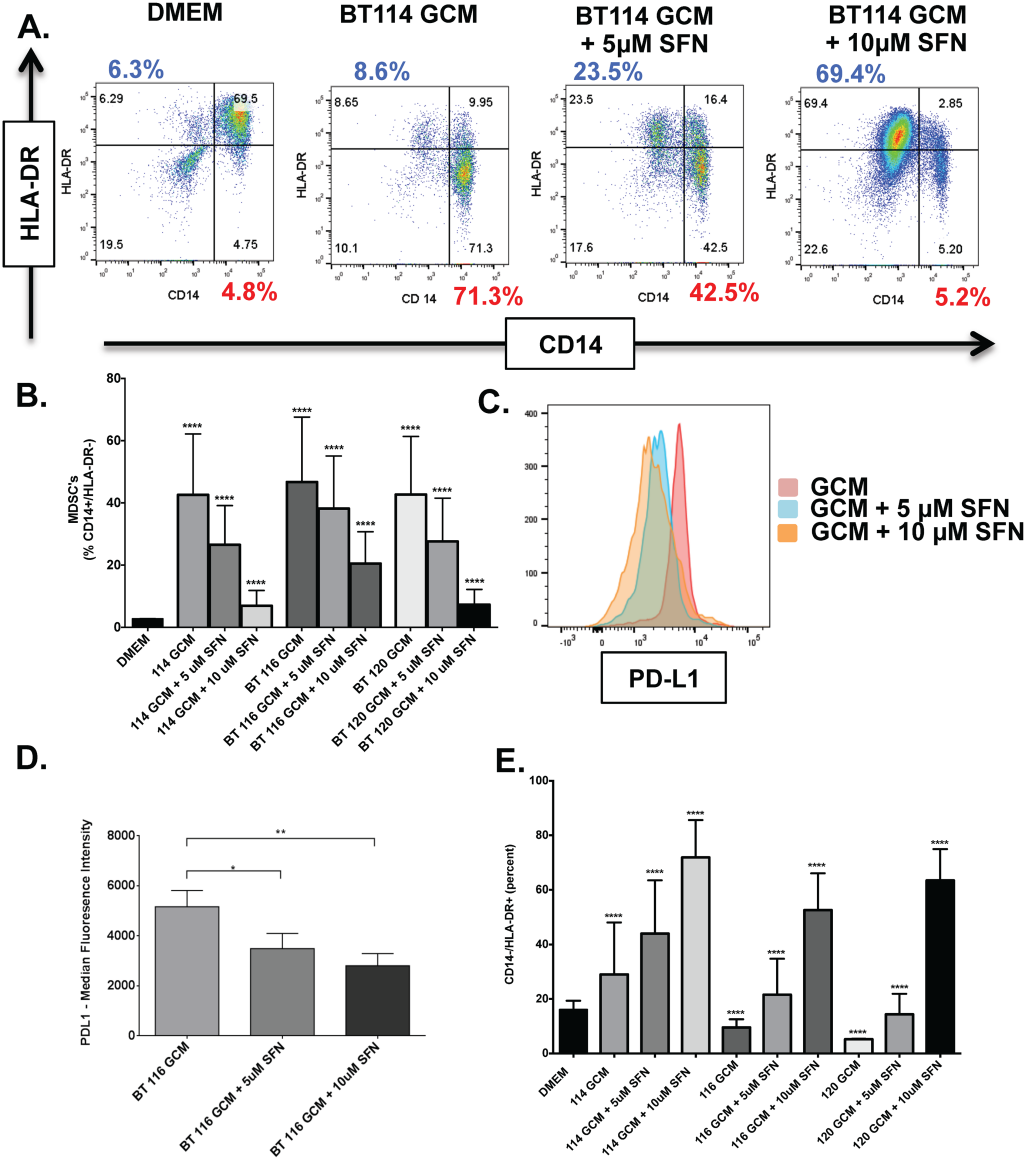
SFN decreases immunosuppressive MDSC formation in response to GCM but increases CD14-/HLA-DR+ cells. All experiments performed under hypoxic (1% O_2_) conditions. A) Representative dot plots showing CD14/HLA-DR expression in CD14+ monocytes cultured alone or in the presence of BT114 GCM +/- SFN. Monocytes cultured alone are largely CD14+/HLA-DR+ with relatively few mMDSC’s (CD14+/HLA-DR-; 4.75%) (top left). Exposure to BT114 GCM causes a marked increase in mMDSC’s (CD14+/HLA-DR-; 71.3%) (top right). Addition of SFN is associated with a dose-dendent reduction in mMDSC’s (5uM SFN = 42.5% mMDSC’s; 10uM SFN = 5.2% mMDSC’s) (bottom left and right; percent shown in red). Interestingly, this reduction in mMDSC’s is associated with an increase in CD14-/HLA-DR+ cells (percent shown in blue). B) SFN dose-dependent reductions in CD14+/HLA-DR- MDSCs in response to three different GCM’s were seen across monocytes from two unique healthy donors. C) Representative histograms showing immunosuppressive PD-L1 expression in monocytes exposed to BT116 GCM that decreases in a dose-dependent fashion with addition of SFN. D) Bar graph showing median fluorescence intensity of PD-L1 expression in monocytes from three donors exposed to BT116 GCM +/- SFN. E) Bar graph showing increasing population of CD14- / HLA-DR+ cells after SFN exposure among CD14+ cells cultured in GCM (from same experiments as 5A-B). * P < 0.05, ** = P< 0.01,**** = P<0.0001.

SFN inhibited the formation of CD14+/HLA-DR- MDSCs in CD14+ monocytes exposed to GCM in a dose-dependent fashion (Fig 5A and 5B). This phenotypic change was significantly inhibited in three monocyte donors at both 5 μM and 10 μM SFN (Fig 5B) (114 GCM vs. 114 GCM+5uM SFN, p<0.05, 95% CI(15.14 to 16.92); 114 GCM vs. 114 GCM+10uM SFN, p <0.05, 95% CI(34.74 to 36.51); 116 GCM vs. 116 GCM+5uM SFN, p<0.05, 95% CI(7.644 to 9.420); 116 GCM vs. 116 GCM+10uM SFN, p<0.05, 95% CI(25.32 to 27.10); 120 GCM vs. 120 GCM+5uM SFN, p<0.05, 95% CI(14.23 to 16.00); 120 GCM vs. 120 GCM+10uM SFN, p<0.05, 95% CI(34.49 to 36.26)). This was associated with decreased expression of the immunosuppressive protein PD-L1 (B7-H1, CD274). Monoclonal antibodies targeting the PD-L1 / PD-1 immune checkpoint are currently revolutionizing systemic cancer treatment [32, 33] but their efficacy for intracranial tumors is less certain given their poor blood-brain barrier penetration. GCM-treated monocytes are generally positive for PD-L1 by flow cytometry (Fig 5C). PD-L1 expression is significantly decreased by SFN exposure in a dose-dependent fashion (5 μM SFN: p = 0.0261, 95% CI 203.9 to 3146; 10 μM SFN: p = 0.0033, 95% CI 888.4 to 3831) (Fig 5D). Finally, we noted that monocytes cultured in GCM with 5uM and 10 uM SFN also skew toward a CD 14-/HLA-DR+ phenotype. (Fig 5A and 5E)

### Sulforaphane increases dendritic cell frequency in cultured monocytes

**Fig 6:**
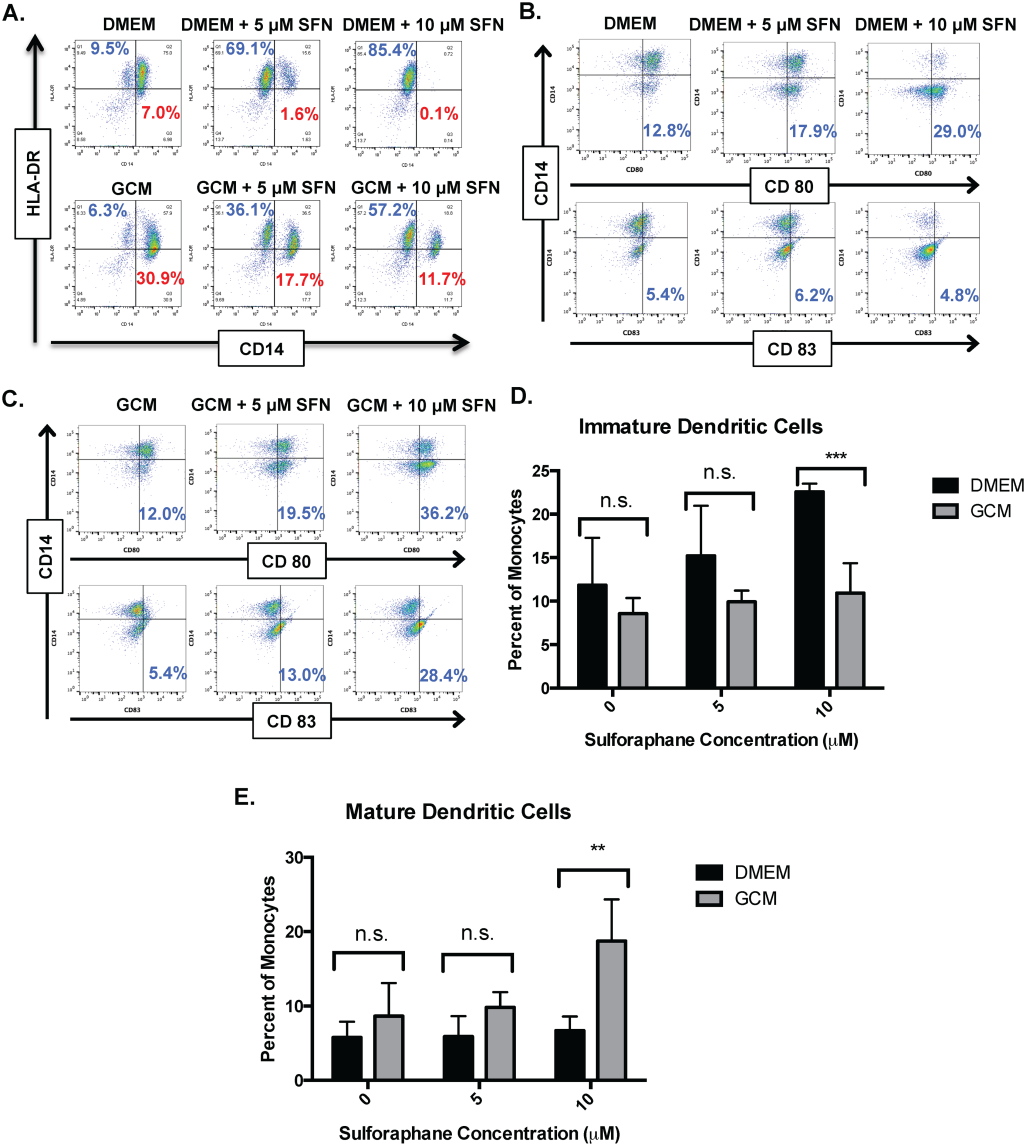
SFN promotes dendritic cell development from monocytes in both fresh media and GCM. All experiments performed under hypoxic (1% O_2_) conditions. A) Representative dot plots showing CD14 and HLA-DR expression in CD14+ monocytes cultured in fresh media (DMEM) or BT116 GCM +/- SFN. Note that in addition to reductions in CD14+/HLA-DR- mMDSC’s (percent shown in red), there is a SFN-dependent increase in CD14- / HLA-DR+ cells suggestive of dendritic cells (percent shown in blue) in both fresh media and to a lesser extent in GCM. B) Representative dot plots showing CD14, HLA-DR, CD80, and CD83 expression in CD14+ monocytes cultured in DMEM +/- SFN. C) Representative dot plots showing CD14, HLA-DR, CD80, and CD83 epxression in CD14+ monocytes cultured in BT116 GCM +/- SFN. D) Bar graphs showing mean frequency of immature dendritic cells (CD14-/HLA-DR+/CD80+/CD83-) after culturing CD14+ monocytes from three donors in fresh media (DMEM) or BT116 GCM +/- SFN. Note that there is a SFN dose-dependent increase in immature dendritic cells with fresh media. E) Bar graphs showing mean frequency of mature dendritic cells (CD14-/HLA-DR+/CD80+/CD83+) after culturing CD14+ monocytes from three donors in fresh media (DMEM) or BT116 GCM +/- SFN. Note that there is a SFN dose-dependent increase in mature dendritic cells with GCM. n.s. = not significant, ** = P<0.01, *** = P<0.001.

We were intrigued by the corresponding increase in CD14-/HLA-DR+ monocytes that occurred in addition to decreases in CD14+/HLA-DR- monocytic MDSC’s following SFN exposure. We found that this occurred both in the presence of fresh media and GCM, though to a greater extent with fresh media (Fig 6A). The accumulation of CD14-/HLA-DR+ cells among cultured CD14+ cells suggested possible conversion to dendritic cells. To test this, we examined CD14, HLA-DR, CD80, and CD83 expression in monocytes cultured in either DMEM (Fig 6B) or GCM (Fig 6C) in the presence of increasing concentrations of SFN. SFN exposure was associated with a significant dose-dependent enrichment in CD14-/HLA-DR+ and CD14-/CD80+ cells from among CD14+ monocytes cultured in either DMEM or GCM in keeping with conversion to dendritic cells. However, expression of the dendritic cell maturation marker CD83 among CD14- cells was only increased in monocytes cultured in GCM. Monocytes cultured in DMEM had a trend to increased immature dendritic cells (CD14-/HLA-DR+/CD80+/CD83-) compared to monocytes cultured in GCM that increased with SFN exposure and was significantly different at 10 μM (Fig 6D). Similarly, monocytes cultured in GCM had a trend to increased mature dendritic cells (CD14-/HLA-DR+/CD80+/CD83+) compared to monocytes cultured in DMEM that increased with SFN exposure and was significantly different at 10 μM (Fig 6E).

### Sulforaphane increases T cell proliferation and overcomes MDSC-mediated T cell inhibition

**Fig 7:**
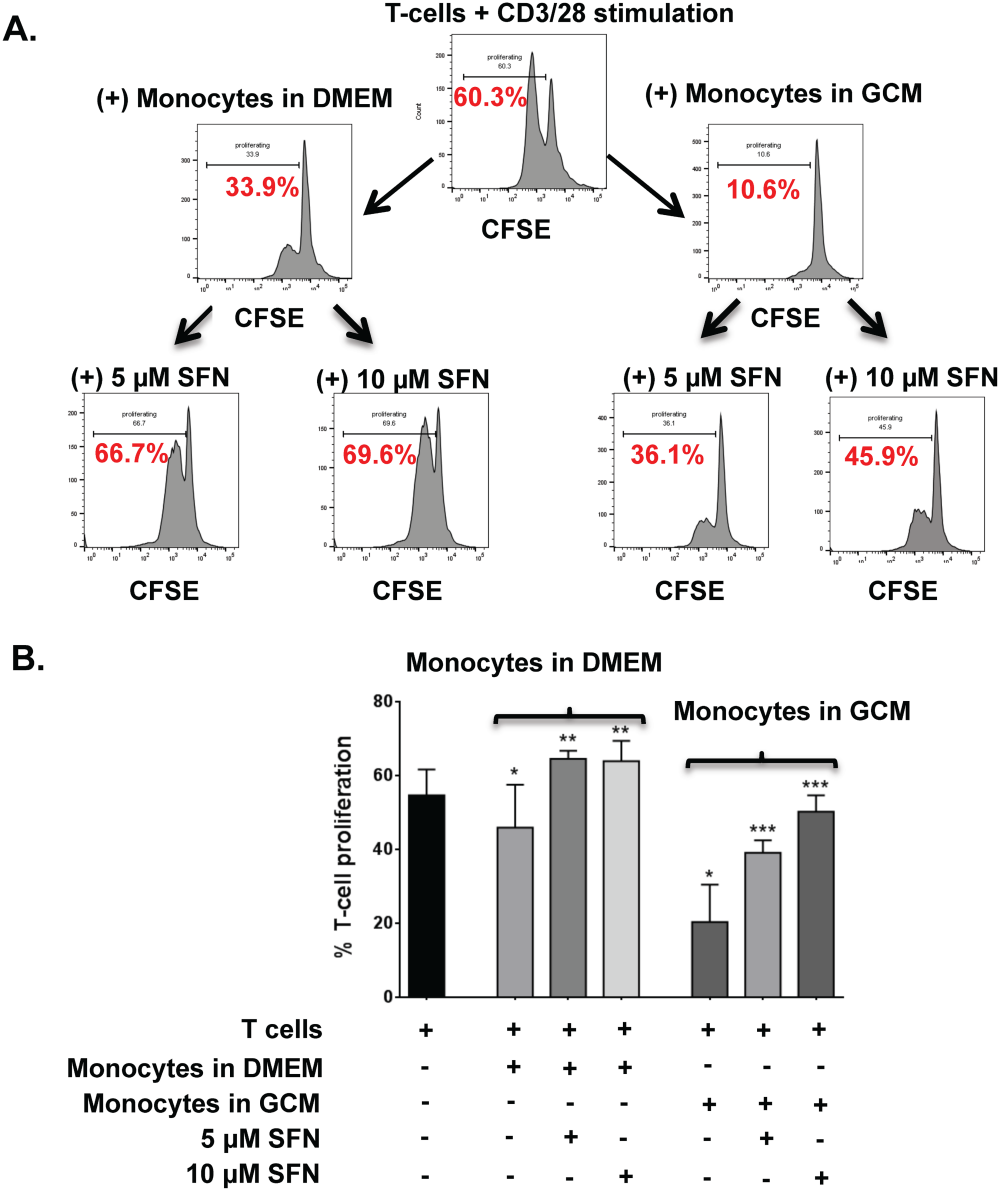
SFN treated monocytes cultured in both fresh media and GCM promote T-cell proliferation. All experiments performed under hypoxic (1% O_2_) conditions. A) An experiment schematic with representative histograms showing proliferation of CFSE stained T-cells stimulated with anti-CD3/anti-CD28 antibodies with or without prior exposure to autologous monocytes cultured in fresh media (DMEM) or BT116 GCM +/- addition of SFN. B) Bar graphs showing mean T cell proliferation (from three donors) in response to anti-CD3 / anti-CD28 antibodies in the conditions outlined. Exposure to monocytes pre-cultured in DMEM caused a modest but statistically significant reduction in proliferation compared to T cells alone. This could be increased to proliferation greater than baseline by additional exposure to SFN. Exposure to monocytes pre-cultured in GCM (i.e. MDSC-enriched) caused a much greater inhibition of T cell proliferation which could be at least partly reversed in a SFN dose-dependent fashion. * P < 0.05, ** P < 0.01, *** P < .001.

CD3+ T cells were stimulated with anti-CD3 / anti-CD28 antibodies in the presence or absence of autologous CD14+ monocytes pre-cultured for 48 hours in either fresh DMEM or GCM with or without increasing concentrations of SFN. T cell proliferation was determined by CFSE-staining (Fig 7A). Both monocytes cultured in GCM (MDSC-enriched) and monocytes cultured in fresh media (low MDSC frequency) decreased CD3+ autologous T cell proliferation in response to anti-CD3 / anti-CD28 beads compared to T cells alone, though this was more marked with GCM-treated monocytes (T-cells alone vs. T-cell + monocytes in fresh media, p<0.05, 95% CI(7.614 to 9.853); T-cells alone vs. T-cells + GCM-treated monocytes, p<0.05, 95% CI(33.15 to 35.39)) (Fig 7B). SFN increased T cell proliferation in response to anti-CD3 / anti-CD28 stimulation in a dose-dependent fashion in the presence of both monocytes cultured in fresh media and monocytes cultured in GCM, though this was more marked for monocytes in fresh media (T-cells + Monocytes precultured in DMEM vs. T-cells + Monocytes precultured in DMEM + 5uM SFN, p <0.05, 95% CI(-19.75 to -17.55); T-cells + Monocytes precultured in DMEM vs. T-cells + Monocytes precultured in DMEM + 10uM SFN, p <0.05, 95% CI(-19.15 to -16.95); T-cells + Monocytes precultured in GCM vs. T-cells + Monocytes precultured in GCM + 5uM SFN, p <0.05, 95% CI(-19.85 to -17.65); T-cells + Monocytes precultured in GCM vs. T-cells + Monocytes precultured in GCM + 10uM SFN, p <0.05, 95% CI(-30.96 to -28.77)) (Fig 7B). In the monocytes pretreated in GCM group, there was a significant increase in T-cell proliferation in the 10uM SFN treated group compared to the 5uM SFN treated group (p<0.05, 95% CI(-12.21 to -10.02)). Both monocyte groups pretreated in DMEM + 5uM SFN (p<0.05, 95% CI(-11.01 to -8.822)) or 10uM SFN (p<0.05, 95% CI(-10.41 to -8.222)) showed increased T-cell proliferation compared to T-cell stimulated alone.

## Discussion

Immunotherapy holds promise for cancer treatment but is limited by tumor-mediated immunosuppression. MDSCs are a major contributor to immunosuppression in many cancers, including GBM. The most accepted understanding of MDSC development in cancer is that normal monocytes enter the tumor microenvironment and are educated through both cell-cell contact with the tumor cells and through secreted factors.[16, 34] The initial transformation is to the monocytic form of MDSCs (mMDSCs) and then later to granulocytic MDSCs.[8] Study of MDSC function in human cancers including gliomas has been slowed by limited availability of patient specimens. Therefore, we sought to develop methods to generate large numbers of MDSCs *in vitro*. We focused on mMDSCs as their surface marker phenotype is easily identifiable using only surface markers and their formation is a critical first step in MDSC development. We have previously generated MDSCs *in vitro* by co-culturing normal monocytes with human GBM cell lines [16, 17]. However, we found this suboptimal for large experiments due to additional variable culture requirements and the need to purify MDSC’s from co-cultures for subsequent analyses.

We have now developed a simple and robust method to generate CD11b+/CD14^+^/HLA-DR^-^ /CD15^-^ monocytic MDSCs from normal monocytes through culture in human glioma-conditioned media and confirmed that the frequency of these cells can be approximated (though slightly over-estimated) by determining the frequency of CD14^+^/HLA-DR^-^ cells (Fig 1). MDSC development is also increased by a hypoxic culture environment which may simulate the tumor microenvironment [26–28]. Finally, lyophilized and reconstituted glioma-conditioned media generated mMDSCs as effectively as fresh glioma-conditioned media. (Fig 1C) This further demonstrates the reproducibility of this technique and highlights the role of soluble tumor-derived factors in generating MDSCs as no glioma cells would have survived lyophylization.

To help determine what soluble factors might be contributing to MDSC generation, we screened GCM from three human glioma cell lines for 51 cytokines using a commercially available array (Table 1). Factors significantly elevated in at least two of three lines included angiogenin, IGFBP 2&3 (insulin-like growth factor binding protein), IL-6, IL-8, MCP-1 (CCL2/monocyte chemoattractant protein-1), MIF (macrophage migration inhibitory factor), osteopontin, and TIMP 1&2 (tissue inhibitor of metalloproteinases). Several pertinent negative findings were also indicated by the cytokine array data. Macrophage Colony-Stimulating Factor (MCSF, also known as colony-stimulating factor 1 or CSF1) and Transforming Growth Factor-β (TGF-β) have both been reported to be expressed by GBMs in the past and both have been associated with MDSC accumulation in cancer.[2, 16, 35–37] However, neither was present above background in our glioma-conditioned media. Similarly, VEGF is known to be secreted by GBMs and has been associated with MDSC accumulation.[6] While it was present in two out of three glioma conditioned medias, the levels of expression were modest.

It is well established that some of the factors identified in our screen of glioma-conditioned media may have roles in MDSC accumulation. IL-6 is expressed by GBMs[19] and has been associated with the accumulation of MDSCs in human cancers and in animal models.[38–40] IL-6 is also a potent activator of STAT3 which, in turn, is associated with MDSC mediated immunosuppression.[41] Similarly, IL-8 expression by GBM’s may be important in trafficking MDSCs into the tumor environment by acting on the CXCR2 receptor [42] (which can also serve as a receptor for MIF [43]). Finally, MCP-1 (CCL-2) showed the highest level of expression across our three glioma conditioned medias. MCP-1 is known to be expressed by GBMs and has been shown to play a crucial role in the migration of MDSCs into the tumor environment as well as in promotion of tumor growth by MDSCs.[17, 44, 45] Other factors present in glioma-conditioned media that could impact MDSC development are less well characterized but not without precedent. For example, osteopontin expression by GBM has been associated with cancer stem cells and tumor-infiltrating macrophages [46, 47]. In other cancers, it has been associated with extramedullary hematopoiesis and expansion of MDSCs in-vivo.[48] Blocking osteopontin in a mouse model led to increase efficacy of a B-cell based tumor vaccine. The roles of TIMP-1 and TIMP 2 (both are inhibitors of matrix metalloproeteinases) in MDSC generation are less clear, though serum TIMP-1 levels are associated with shorter survival in many types of cancer [49] and TIMP-2 has been shown to be a negative regulator of MDSCs in mice.[50]

Our attention was particularly drawn to Macrophage Migration Inhibitory Factor (MIF), which was significantly elevated across all three of our glioma conditioned medias. MIF expression in GBM has been previously reported [51, 52], but its role in GBM-mediated immunosuppression is only beginning to be elucidated [53]. However, in other tumors MIF secretion is associated with increased intratumoral mMDSCs [54] and adding an inhibitor of MIF’s keto-enol tautomerase activity to tumor supernatant has decreased the formation of mMDSCs.[55] One group has gone as far as to call MIF, “MDSC inducing factor”, due to its ability to increase mMDSCs in the tumor microenvironment.[56] Otvos et al have recently described MIF’s role in generating MDSCs in both murine glioma models and patient-derived specimens [57]. Interestingly, they found that MIF production was limited to cancer stem cells, not differentiated glioma cells. In our hands, differentiated human glioma cell lines also secreted abundant MIF. This may reflect some residual “stemness” of these lines as they were originally established as glioma stem cells and only subsequently differentiated by the addition of 10% calf serum [58]. Our results showed that MIF was necessary for MDSC induction by glioma-conditioned media but not sufficient. It is possible that other factors contained within GCM like IL-6 (which has been associated MDSC accumulation in some reports [38]) may be necessary as well.

Thus, MIF is an attractive therapeutic target to reverse glioma-mediated MDSC accumulation. We sought an agent with good oral bioavailability, safety proflile, and blood-brain barrier penetration. Sulforaphane (SFN), a phytochemical readily available in broccoli sprouts, potently inhibits MIF’s enzymatic activity. Sulforaphane’s bioavailability through oral intake is excellent [59, 60]. SFN has been investigated in multiple clinical trials for a variety of indications including autism and schizophrenia. It has good blood-brain barrier penetration and it is possible to reach therapeutic concentrations *in situ* via oral intake [61]. It is remarkably non-toxic to healthy cells[62–64] but has been reported to have direct anti-tumor effects on cancer cells [65] [66, 67]. To our knowledge, SFN’s ability to modulate glioma-mediated immunosuppression has not been investigated previously. We found that addition of anti-MIF blocking antibody blocks the formation of CD14+/HLA-DR- MDSC’s induced by glioma-conditioned media in our model system (Fig 2). GCM has intrinsic tautomerase activity compatible with the presence of MIF that is inhibited by the addition of SFN (Fig 3). SFN is non-toxic to leukocytes at moderate to high concentrations but shows some intrinsic anti-glioma properties at lower concentrations (Fig 4). Furthermore, SFN at relatively low concentrations reduces MDSC formation in glioma-conditioned media (Fig 5). Previous studies have demonstrated an upregulation of PD-L1 on MDSCs under hypoxic conditions which further increases the immunosuppressive ability of these cells and reflecting HIF1α expression in the tumor microenvironment.[68] [69] Our data corroborates this showing relatively high levels of PD-L1 expression in mMDSCs which is then decreased upon exposure to SFN. (Fig 5C and 5D)

Interestingly, in addition to its effects reducing MDSC accumulation, SFN promotes the development of pro-inflammatory dendritic cells from monocytes cultured in both fresh media and glioma-conditioned media, though these are only mature DC’s (CD83+) in the presence of GCM (Fig 6). Limited data regarding the impact of SFN on dendritic cells has been reported previously. Much like our findings, Singh et al found that SFN could decrease murine prostate carcinoma growth and metastasis via proinflammatory effects on both dendritic cells and NK lymphocytes [70]. Similarly, Kim et al reported that SFN reversed age-related decreases in TH1 responses in mice via pro-inflammatory effects on dendritic cells [71]. However, other studies have provided conflicting findings. Geisel et al found that oral SFN administration protected mice from experimental allergic encephalomyelitis by inhibiting TLR4-induced IL-12 and IL-23 secretion by dendritic cells [72]. Qu X, et al reported that SFN inhibited maturation of porcine dendritic cells induced by lipopolysacharride [73]. Although the impact of SFN on decreasing MDSC development has appeared tightly linked to its ability to inhibit MIF function[55], the mechanisms invoked for these diverse effects on dendritic cell development have been more varied and include epigenetic modification through histone deacetylase (HDAC) inhibition, decreased NFκB transcription, and NRF2-mediated increases in anti-oxidants. Whether, these effects are context and/or species-dependent remains to be seen. Keeping with the apparently different mechanisms underlying SFN’s effects on MDSC and DC development, we found that anti-MIF antibody treatment markedly reduced MDSC formation but only modestly increased the frequency of CD14-/HLA-DR+ cells (i.e. dendritic cells; Fig 2). In contrast, SFN treatment resulted in both marked decreases in MDSC’s and increases in dendritic cells (Figs 5 and 6). Regardless, the pro-inflammatory effects of SFN on MDSC’s and DC’s in our *in vitro* glioma culture systems suggests that it may be an attractive immunomodulatory agent in glioma treatment.

In addition to its pro-inflammatory effects, SFN was also directly toxic to glioma cells suggesting that it might have additional therapeutic benefits. The mean IC50 of SFN for 3 different glioma cells lines was 17.29 μM. This is higher than the concentrations needed to induce pro-inflammatory effects (5 μM– 10 μM) but substantially lower than concentrations that induce toxicity in normal monocytes and lymphocytes (55.63 μM– 80.22 μM). Serum SFN concentrations between 15 μM– 20 μM are obtainable with oral intake.[74] However, it remains uncertain how much SFN crosses the blood brain barrier and a chemotherapeutic dose of SFN via oral intake may be difficult to achieve for central nervous system tumors.[75] However, several smaller clinical trials have been published with evidence that SFN is beneficial in treatment of central nervous system disorders such as autism and schizophrenia [76, 77]. Both of these studies suggested that SFN’s predominant mechanism of action in these conditions is protection against oxidative stress. It is not clear if this occurs within the CNS due to direct action of SFN in the brain or if systemic administration of SFN results in a systemic reduction of oxidative stress and a secondary reduction in reactive oxygen species entering the CNS. Similarly, we would predict that orally administered SFN may have efficacy against brain tumors like gliomas through a secondary reduction in systemic immunosuppression. Whether it would also have direct anti-glioma properties against these intracranial tumors is less certain.

Other MIF inhibitors have been described including the small molecule 4-iodo-6-phenylpyrmidine (4-IPP) which has been reported to inhibit MDSC development [78, 79]. It will be interesting to compare results of MIF inhibition with SFN to agents like 4-IPP in the future. Results of our tautomerase inhibition assay showed marked inhibition with 5–10 μM SFN which is similar or possibly more potent that reported results with 4-IPP. Further testing will be required to compare their activity directly, relative specificity, and pharmacokinetic properties.

## Conclusion

Understanding the mechanisms of glioblastoma and cancer induced immunosuppression are key to developing effective immunotherapies. In this study, we have demonstrated a simple method for generating large amounts of immunosuppressive MDSCs by culturing normal monocytes in glioma-conditioned media. This method can be used for further mechanistic studies to understand the factors driving MDSC function and accumulation. Our cytokine array data suggests a number of potential secreted factors that may play a role in MDSC accumulation in GBM. MIF, an abundant cytokine in three unique human glioma conditioned medias, may play a role in mMDSC formation as an MIF neutralizing antibody negates the effect of GCM on monocytes. MIF has tautomerase activity which has been linked to its functional activity and is inhibited by the phytochemical sulforaphane. Sulforaphane inhibits the formation of mMDSCs *in vitro* when monocytes are exposed to glioma conditioned media, decreases their expression of immunosuppressive PD-L1, and drives them towards a mature dendritic cell phenotype which promotes T-cell proliferation. Sulforaphane has additional activity directly against glioma cells directly at concentrations that are not toxic to normal monocytes and lymphocytes and can be achieved in serum through oral administration. Sulforaphane is available through a normal diet from cruciferous vegetables, can be administered orally as a supplement or drug, and has low toxicity in humans. It is thus attractive for further investigation in GBM patients as an immunomodulatory agent and/or for its direct anti-tumor effects.

## Supporting information

S1 FigFlow cytometry gating strategy for M-MDSC’s and G-MDSC’s.Representative histograms and dot plots showing the flow cytometry gating strategy used to define M-MDSC’s (CD11b^+^/CD14^+^/CD15^-^/HLA-DR^LO/-^) and G-MDSC’s (CD11b^+^/CD14^-^/CD15^+^) among healthy donor monocytes cultured with serum-free media (top row) or glioma-conditioned media (bottom row). The first histogram shows %CD11b^+^ among all cells, the next dot plot shows CD14 / HLA-DR expression among CD11b^+^ cells, the subsequent dot plot shows CD15^+^ frequency among “R1” (CD11b^+^/CD14^-^), and the final dot plot shows CD15^-^ frequency among “R2” (CD11b^+^/CD14^+^/HLA-DR^LO/-^). MDSC frequency as a percentage of CD11b+ cells is determined by the multiplication of the frequency of the appropriate subsets. This can be defined as follows for the representative cases shown:
$$\begin{array}{ll} \text{M}-\text{MDSC}’\text{s} & =\text{R}2\;\left(\text{CD}14+/\text{HLA}-{\text{DR}}^{\text{LO}/-}\right)\text{ as }\%\text{CD}11{\text{b}}^{+}\text{ x Fraction of CD}15^{-}\text{ in R}2\\ & =1.42\%\text{ x }0.723\;=\;1.02\%\;\left(\text{serum}-\text{free media alone}\right)\\ & =13.9\%\text{ x }0.656\;=\;9.12\%\;\left(\text{glioma}-\text{conditioned media}\right) \end{array}$$
$$\begin{array}{ll} \text{G}-\text{MDSC}’\text{s} & =\text{R}1\;\left(\text{CD}14^{-}\right)\text{ as}\%\text{CD}11{\text{b}}^{+}\text{ x Fraction of CD}15^{+}\text{ in R}1\\ & =85.2\%\text{ x }0.166\;=\;14.14\%\;\left(\text{serum}-\text{free media alone}\right)\\ & =67.0\%\text{ x }0.069\;=\;4.62\%\;\left(\text{glioma}-\text{conditioned media}\right) \end{array}$$(TIFF)

S2 Fig MIF ELISA standard curve. Graph demonstrating the optical density at 450 nm over a range of known MIF concentrations (0.156 ng/mL– 10 ng/mL). This was performed with the supplied MIF and reagents per the kit manufacturer’s protocol. Note that all glioma-conditioned media samples required 1:10 dilution in PBS to reach concentrations that produced optical densities between 0.1 and 1.0. There has been correction for this dilution factor in the MIF ELISA results presented in Fig 2A.
(TIFF)
