# Supplementary figures and images for: Modulating glioma-mediated myeloid-derived suppressor cell development with sulforaphane
Source: PLoS One. 2017 Jun 30;12(6):e0179012. doi: 10.1371/journal.pone.0179012 (PMC5493295; doi:10.1371/journal.pone.0179012)

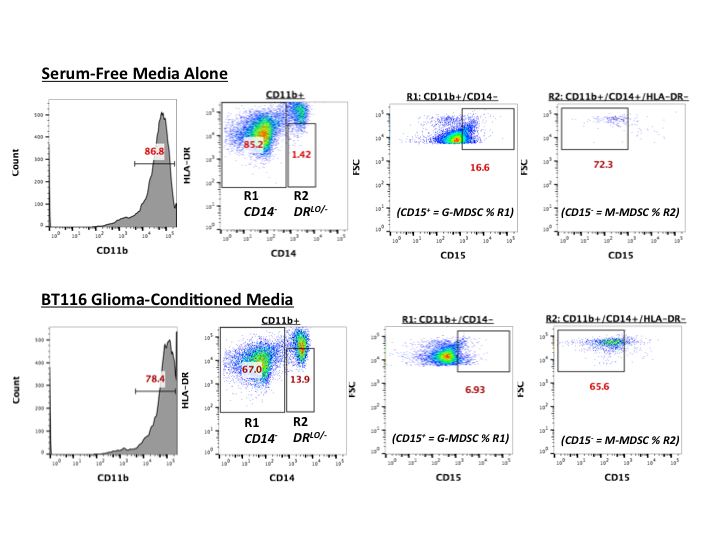

Supplement: S1 Fig — Representative histograms and dot plots showing the flow cytometry gating strategy used to define M-MDSC’s (CD11b+/CD14+/CD15-/HLA-DRLO/-) and G-MDSC’s (CD11b+/CD14-/CD15+) among healthy donor monocytes cultured with serum-free media (top row) or glioma-conditioned media (bottom row). The first histogram shows %CD11b+ among all cells, the next dot plot shows CD14 / HLA-DR expression among CD11b+ cells, the subsequent dot plot shows CD15+ frequency among “R1” (CD11b+/CD14-), and the final dot plot shows CD15- frequency among “R2” (CD11b+/CD14+/HLA-DRLO/-). MDSC frequency as a percentage of CD11b+ cells is determined by the multiplication of the frequency of the appropriate subsets. This can be defined as follows for the representative cases shown: M-MDSC’s=R2 (CD14+/HLA-DRLO/-) as %CD11b+ x Fraction of CD15- in R2=1.42% x 0.723 = 1.02% (serum-free media alone)=13.9% x 0.656 = 9.12% (glioma-conditioned media) G-MDSC’s=R1 (CD14-) as%CD11b+ x Fraction of CD15+ in R1=85.2% x 0.166 = 14.14% (serum-free media alone)=67.0% x 0.069 = 4.62% (glioma-conditioned media) (TIFF) [file pone.0179012.s001.tiff]

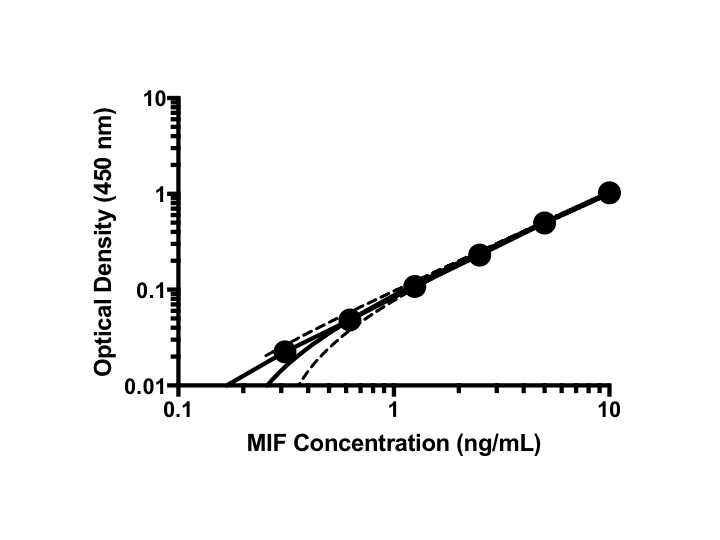

Supplement: S2 Fig — Graph demonstrating the optical density at 450 nm over a range of known MIF concentrations (0.156 ng/mL– 10 ng/mL). This was performed with the supplied MIF and reagents per the kit manufacturer’s protocol. Note that all glioma-conditioned media samples required 1:10 dilution in PBS to reach concentrations that produced optical densities between 0.1 and 1.0. There has been correction for this dilution factor in the MIF ELISA results presented in Fig 2A. (TIFF) [file pone.0179012.s002.tiff]
